# Enhanced Protective Efficacy of a Chimeric Form of the Schistosomiasis Vaccine Antigen *Sm*-TSP-2

**DOI:** 10.1371/journal.pntd.0001564

**Published:** 2012-03-13

**Authors:** Mark S. Pearson, Darren A. Pickering, Henry J. McSorley, Jeffrey M. Bethony, Leon Tribolet, Annette M. Dougall, Peter J. Hotez, Alex Loukas

**Affiliations:** 1 Queensland Tropical Health Alliance and School of Public Health and Tropical Medicine, James Cook University, Cairns, Queensland, Australia; 2 Department of Microbiology, Immunology and Tropical Medicine, George Washington University, Washington D.C., United States of America; 3 Department of Pediatrics and Molecular Virology and Microbiology, and National School of Tropical Medicine, Sabin Vaccine Institute and Texas Children's Hospital Center for Vaccine Development, Baylor College of Medicine, Houston, Texas, United States of America; Institute of Tropical Medicine (NEKKEN), Japan

## Abstract

The large extracellular loop of the *Schistosoma mansoni* tetraspanin, *Sm*-TSP-2, when fused to a thioredoxin partner and formulated with Freund's adjuvants, has been shown to be an efficacious vaccine against murine schistosomiasis. Moreover, *Sm*-TSP-2 is uniquely recognised by IgG_1_ and IgG_3_ from putatively resistant individuals resident in *S. mansoni* endemic areas in Brazil. In the present study, we expressed *Sm*-TSP-2 at high yield and in soluble form in *E. coli* without the need for a solubility enhancing fusion partner. We also expressed in *E. coli* a chimera called *Sm*-TSP-2/5B, which consisted of *Sm*-TSP-2 fused to the immunogenic 5B region of the hookworm aspartic protease and vaccine antigen, *Na*-APR-1. *Sm*-TSP-2 formulated with alum/CpG showed significant reductions in adult worm and liver egg burdens in two separate murine schistosomiasis challenge studies. *Sm*-TSP-2/5B afforded significantly greater protection than *Sm-*TSP-2 alone when both antigens were formulated with alum/CpG. The enhanced protection obtained with the chimeric fusion protein was associated with increased production of anti-*Sm*-TSP-2 antibodies and IL-4, IL-10 and IFN-γ from spleen cells of vaccinated animals. Sera from 666 individuals from Brazil who were infected with *S. mansoni* were screened for potentially deleterious IgE responses to *Sm*-TSP-2. Anti-Sm-TSP-2 IgE to this protein was not detected (also shown previously for *Na*-APR-1), suggesting that the chimeric antigen *Sm*-TSP-2/5B could be used to safely and effectively vaccinate people in areas where schistosomes and hookworms are endemic.

## Author Summary

There are currently no vaccines available to combat helminth (worm) infections in humans. The most devastating of the diseases caused by human helminths are schistosomiasis (or bilharzia) and hookworm disease. By fusing one of the lead schistosomiasis vaccine antigens, *Sm*-TSP-2, with a protective fragment from one of the lead hookworm vaccine antigens, *Na-*APR-1, we have produced a chimeric vaccine, termed *Sm-*TSP-2/5B that might provide protection against two debilitating and co-endemic neglected tropical diseases. *Sm*-TSP-2/5B provided increased protection compared to *Sm*-TSP-2 alone when formulated with human approved adjuvants and tested in a mouse model of schistosomiasis. Moreover, IgE against *Sm*-TSP-2 or *Na-*APR-1 has not been detected in the blood of residents from an area in Brazil that is endemic for schistosomes and hookworms, indicating that vaccines based on these molecules would be unlikely to generate allergic reactions in recipients from developing countries.

## Introduction

Schistosomiasis ranks among the most important infectious diseases in tropical regions, resulting in a loss of between 4.5 and 92 million Disability-Adjusted Life Years (DALYs) annually and almost 300,000 deaths in sub-Saharan Africa alone [1], [2], [3]. High rates of post-treatment reinfection [1], the inability of periodic chemotherapy to interrupt transmission [4], the exclusive reliance on praziquantel as the only chemotherapeutic option [5], [6] and the unsustainability of mass drug administration [7] has led to the development of new anti-schistosomiasis control measures, inlcuding vaccines, to complement existing initiatives [5], [8], [9].

Molecules lodged in the apical membrane of the schistosome tegument represent vulnerable targets for immunological attack by host antibodies due to their intimate association with the host immune system. One such family of molecules – predicted by proteomic analyses of the schistosome tegument to be accessible to host immunoglobulin [10] – is the tetraspanin integral membrane proteins. Tetraspanins contain four transmembrane domains and two extracellular loops that are predicted to interact with exogenous ligands [11], [12]. Indeed, the second extracellular loop of one of these schistosome tetraspanins, *Sm*-TSP-2, has proven to be an effective anti-schistosomiasis vaccine, eliciting 57–64% protection in mice vaccinated with the antigen followed by challenge with *S. mansoni* cercariae [12]. Other schistosome tetraspanins are protective in mouse models of schistosomiasis [10], including *Sm*23 [13], [14] and *Sj-*TSP-2, an *S. japonicum* orthologue of *Sm-*TSP-2 [15]. Moreover, *Sm*-TSP-2 was strongly recognised by IgG1 and IgG3 from putatively resistant but not from chronically infected individuals [12], further highlighting the promise of this antigen as a subunit vaccine against human schistosomiasis.

The tegument of adult and schistosomula of *S. mansoni* is thinner and distinctly more vacuolated compared to controls after *in vitro* treatment with *Sm-tsp-2* double-stranded RNA (dsRNA) [16]. Moreover, injection of mice with schistosomula pre-treated with *Sm-tsp-2* dsRNA resulted in the recovery of 83% fewer parasites from the mesenteries compared to controls [16], highlighting the importance of *Sm*-TSP-2 in proper tegument development and worm survival, and providing a potential mechanism by which the vaccine exerts its protective effect.

In an earlier study, we reported the production of a chimeric form of *Sm*-TSP-2, consisting of *Sm*-TSP-2 fused to the immunodominant and neutralizing 5B region of the hookworm aspartic protease *Na*-APR-1, termed *Sm*-TSP-2/5B [17]. Hookworm infection and schistosomiasis caused by *S. mansoni* are co-endemic in much of sub-Saharan Africa and Brazil, and there is potential interest in developing a vaccine that targets both of these high prevalence and high disease burden helminths [18]. *Na*-APR-1/5B is a 40 amino acid fragment of the protease that contains an immunodominant alpha helix, A_291_Y, which is the target epitope recognized by polyclonal and monoclonal antibodies that are capable of neutralizing the catalytic activity of *Na-*APR-1 [17]. *Na*-APR-1/5B could not be produced in soluble form, but when fused to *Sm*-TSP-2, it was produced in soluble form by *E. coli* and induced antibodies upon vaccination that neutralized the enzymatic activity of *Na*-APR-1; the chimera is currently under investigation as a hookworm vaccine. Using a mouse model of schistosomiasis, we explored the efficacy of the *Sm*-TSP-2/5B chimera in comparison to *Sm*-TSP-2 alone when both antigens are formulated with alum/CpG. Given the recent safety concerns of helminth vaccines that elicit an IgE response in individuals residing in an endemic area [19], we also assessed the recognition of *Sm*-TSP-2/5B by IgE from individuals chronically infected with *S. mansoni*, a crucial step in determining whether or not this antigen could be used to safely and effectively vaccinate people in areas endemic for both hookworms and schistosomes.

## Methods

### Ethics Statement

All work involving experimental procedures with laboratory animals was approved by the animal ethics committee of James Cook University according to the regulations of the Australian Code of Practice for the Care and Use of Animals for Scientific Purposes, 7^th^ edition (reference EA16). All work involving human subjects research was approved by the Human Research Ethics Committees or Institute Review Boards of Instituto René Rachou-FIOCRUZ, the Brazilian National Committee for Ethics in Research (CONEP), George Washington University Medical Center, and the London School of Hygiene and Tropical Medicine. Informed written consent was obtained from all adults or the parents and guardians of all children involved in the study.

### Antigen production

Oligonucleotide primers incorporating *Nde*I and *Xho*I restriction sites (forward primer: GCGCATATGGAAAAGCCCAAGGTCAAAAAACAC; reverse primer GCGCTCGAGGTGCGCTTTGCTTAGATCGCTGAC) and *pfu* turbo DNA polymerase (Stratagene) were used to amplify the extracellular loop 2 region (Glu-107 – His-184) of the *S. mansoni* tetraspanin *Sm*-TSP-2 from the pBAD/TOPO/*Sm*-TSP-2 plasmid [12] in our laboratory. The amplicon was then cloned into the *Nde*I and *Xho*I sites of the pET41a expression vector (Novagen), removing the GST fusion tag to allow for native N-terminal expression of the protein, but retaining the vector's C-terminal 6×his tag to facilitate purification by Immobilised Metal Affinity Chromatography (IMAC). The ensuing plasmid was then transformed into chemically competent *E. coli* BL21-AI cells (Invitrogen). *Sm*-TSP-2 was expressed using the auto-induction method and media formulations established by Studier [20]. Briefly, 10 ml of minimal media supplemented with 50 µg/ml kanamycin (MDG_kan_) was inoculated with a single, recombinant BL21-AI colony and grown overnight at 37°C with shaking (225 rpm). The entire overnight culture was then used to seed 1.0 L of defined media supplemented with 50 µg/ml kanamycin (ZYM-5052-A_kan_), which was incubated for 24 hours at 37°C with shaking (225 rpm). Bacteria were pelleted, lysed and the resultant homogenate purified by (IMAC) as described previously [17]. Purified *Sm*-TSP-2 was buffer-exchanged in a dialysis bag (Pierce) with a cut-off size of 3 kDa against two changes of 50 mM sodium phosphate, pH 6.5, 10 mM NaCl (CEX buffer) (2.0 L each) at 4°C for at least 2 hours and then further purified by passing through a pre-packed 5.0 ml Hi-Trap SP-FF column (GE Healthcare) (equilibrated with 10 column volumes of CEX buffer) at a flow rate of 1.0 ml/min using an AKTA Prime UPC FPLC unit (GE Healthcare). Bound protein was purified by washing with resuspension buffers containing a rising concentration (10–500 mM) of NaCl and eluting in 5 column volumes of elution buffer (50 mM sodium phosphate, pH 6.5, 1.0 M NaCl). *Sm*-TSP-2 was desalted in a dialysis bag (Pierce) with a cut-off size of 3 kDa against two changes of PBS (2.0 L each) at 4°C for at least 2 hours and the final protein concentration was adjusted to 1.0 mg/ml using an Amicon Ultra-15 centrifugal concentration device (Millipore).

*Sm*-TSP-2/5B was produced in *E. coli* and purified as previously described [17]. The pMal-4E plasmid encoding Maltose Binding Protein (MBP) was kindly provided by Dr F. Cardoso and MBP was expressed in *E. coli* and purified on amylose resin according to the manufacturer's instructions (New England Biolabs).

### Generation of rabbit antisera

An emulsion containing 100 µg of *Sm-*TSP-2 or *Sm*-TSP-2/5B (1.0 mg/ml) and an equal volume of Freund's complete adjuvant was subcutaneously injected into a single New Zealand White rabbit. The same amount of antigen emulsified in an equal volume of Freund's incomplete adjuvant was similarly administered 2 and 4 weeks later. The rabbit was bled 2 weeks later and the serum collected by centrifugation.

### Recognition of parasite-derived *Sm-*TSP-2 using rabbit antisera

Freshly perfused adult *S. mansoni* were fixed in 100% methanol overnight at 4°C, embedded in Tissue-tek Optimal Cutting Temperature compound (ProSciTech) and cryostatically sectioned into 7.0 µm sections. Sections were rehydrated in PBS and blocked with PBS/0.05% Tween 20 (PBST)/1% Foetal Calf Serum (FCS) for 1 hour at RT. After washing twice (5 minutes each) with PBST, sections were incubated with either anti-*Sm*-TSP2, anti-*Sm*-TSP2/5B or naive rabbit sera (8.0 µl in 200 µl PBST/1% BSA) and 5.0 µl methanolic Alexa Fluor 488-Phalloidin (Invitrogen) for 1 hour at RT and then washed again (3×5 minutes each). The sections were then probed with goat anti-rabbit IgG-Cy3 (Jackson) (1∶500 in PBST/1% BSA) for 1 hour at RT. After a further 3 washes with PBST, slides were air dried briefly and mounted with cover slips using PBS/50% Vectorshield mounting medium with DAPI (Vector Industries) to stain nuclei. These were examined using a Leica IM1000 DMIRB inverted fluorescence microscope.

### Neutralization of hemoglobinolysis by anti-*Sm*-TSP-2/5B antibodies

The inhibition of hemoglobin digestion by *Na-*APR-1 using anti-*Sm*-TSP2/5B IgG was performed as described previously [17]. An equal amount of anti-*Sm*-TSP-2 IgG was used as a negative control.

### Study population

The study was conducted in Americaninhas, a rural community in northeast Minas Gerais state, Brazil and has been described in detail [12]. The study design was a total population survey, with all individuals in a 10 km^2^ area eligible for inclusion. All participants excluded from the study were offered a fecal exam and treated for all helminth infections, but were not considered part of the data set for analysis. Women who were evidently pregnant, or who tested positive on a urine pregnancy test received treatment for all helminth infections after the end of the pregnancy or the termination of breast-feeding.

### Parasitological survey

The parasitological survey and blood draw were performed during April-July 2004, the results of which can be found in Table 1. Subjects were asked to provide two fecal samples on two separate days, which were examined qualitatively by formalin-ether sedimentation. Helminth-positive samples were then examined by Kato–Katz fecal thick smear to quantify the intensity of infection, as eggs per gram of feces (epg). Two slides were counted from each day's sample, i.e. 2–4 slides from each individual, as some individuals only provided one sample. Individuals who were egg-positive by sedimentation but negative by Kato-Katz were assigned a count of 3 epg, half the Kato-Katz detection limit. Hookworm was exclusively *N. americanus*. Adults or children positive for gastrointestinal nematodes were offered a single 400 mg dose of albendazole and individuals infected with *S. mansoni* were treated with praziquantel. Egg-negative individuals were not treated. Treated individuals were examined post-treatment to confirm treatment efficacy, and offered repeat treatment(s) until egg-negative.

**Table 1:** Cohort details of chronically infected individuals used in this study.

Group	N	Age			*Necator americanus*					*Schistosoma mansoni*				
					Prev.^a^	Intensity of Infection (EPG^b^)				Prev.^a^	Intensity of Infection (EPG^b^)			
		Mean	Min	Max	N (%)	Min	Max	G. Mean	95% CI	N (%)	Min	Max	G. Mean	95% CI
Females	309	33.78	1	96	248 (69)	6	62,856	766	(606, 969)	170 (55)	6	4074	93.33	(74, 116)
Males	357	34.30	7	92	252 (82)	6	55,554	692	(559, 857)	155 (43)	6	5952	90.82	(71, 114)
Total	666	33.45	7	92	500 (75)	6	62,856	727	(621, 858)	325 (49)	6	5952	92.12	(78, 108)

a “Prev.” = prevalence of infection.

b “EPG” = eggs per gram of feces as etermined by 2 slides of Kato Katz fecal thick smear for two days.

### Determination of human IgE responses against *Sm*-TSP-2

Approximately 20 ml of blood was collected from 666 volunteers in siliconized tubes for separation of serum. In brief, the level of IgE against *Sm*-TSP-2 was measured by indirect ELISA using Polysorp 96-well microtiter ELISA plates (NUNC F96, Fisher Scientific) which were incubated overnight at 4°C with antigen (1 µg/ml in 0.15 M PBS, pH 7.2). After washing with PBST, the plates were blocked for 2 hours at RT with 250 µl of PBST/3% BSA. One hundred microliters of sera (1∶25 in PBST/3% BSA) were added to the wells and incubated overnight at 4°C, then the plates were washed with PBST and 100 µl of mouse biotin-conjugated monoclonal anti-human IgE FC (Human Reagent Laboratory, Baltimore, MD) (1∶200 in PBST/3% BSA) was added to the plates. Plates were incubated for 2 hours at RT and then washed with PBST. Plates were developed by adding o-Phenylenediamine dihydrochloride in 0.05 M phosphate-citrate buffer (pH 5.0) plus 30% hydrogen peroxide H_2_O_2_ for 30 minutes at RT in the dark. Fifty microliters of 2N H_2_SO_4_ was added to stop the colorimetric reaction, which was read at a wavelength of 490 nm on a SpectraMax 340 PC (Molecular Devices) microplate reader. SOFTmax Pro for Windows was used for the analysis and storage of data.

### Mouse immunisation and parasite challenge

Approval for the work described in this study was obtained from the James Cook University Animal Ethics Committee. Groups of ten female C57BL/6 mice were immunised with *Sm*-TSP-2, *Sm*-TSP-2/5B, or the control protein MBP. Each antigen (25 µg per dose) was formulated with an equal volume (25 µl) of a 13 mg/ml colloidal suspension of aluminium hydroxide gel (alum) (Sigma) and 5 µg of CpG oligodinucleotide 1826 (CpG) (Invivogen) and injected intraperitoneally on days 0, 14 and 28. Mice were challenged on day 42 with 120 *S. mansoni* cercariae by abdominal penetration [21]. Trials were conducted twice on different dates and with different batches of cercariae. Serum samples were collected at day −2 (pre-immunisation), day 40 (pre-challenge) and day 91 (necropsy) to assess antibody responses.

### Necropsy and estimation of parasite burden

Mouse necropsy and worm and egg burden assessments were performed as described previously [12]. Reductions in parasite loads were calculated as percentages of the parasite burden in the control group. Statistical significance was assigned a threshold of *P* = 0.05 and values were determined using the student's *t* test function in Graph Pad Prism.

### ELISA using pre-challenge and necropsy sera

Individual anti-*Sm*-TSP-2 titres (total IgG, IgG_1_ and IgG_2a_) were determined for all trial 1 animals just prior to cercarial challenge and at necropsy using standard ELISA techniques. Antigen was coated on microtiter plates at 1.0 µg/ml. Sera were serially diluted (1∶1,000 to 1∶16,384,000 for total IgG and IgG_1_ measurements and 1∶1,000 to 1∶256,000 for IgG_2a_ assessment) and 100 µl was added to each well. After addition of the appropriate horseradish peroxidase-conjugated goat antibody (Jackson), peroxidase activity was detected with tetramethyl benzidine chromogenic substrate and measured at 655 nm.

### Cytokine ELISAs with restimulated splenocytes

Spleens were taken from all animals from trial 2, and single cell suspensions prepared by passing through a 70 µm filter (BD Biosciences). Red blood cell lysis buffer (Sigma) was used to remove red blood cells. Splenocyte preparations were counted, and cultured in duplicate at 1×10^6^ cells/well in 96-well plates. Schistosome egg antigen (SEA) and soluble adult worm antigen preparation (SWAP) were prepared as described respectively [18], [19] and added to the cultures at 10 µg/ml and cultured at 37°C, 5% CO_2_ for 72 h. Levels of IL-4, IL-10, and IFN-γ in cell-free supernatants were assessed by ELISA (OptEIA, BD Biosciences).

## Results

### Antigen production

**Figure 1:**
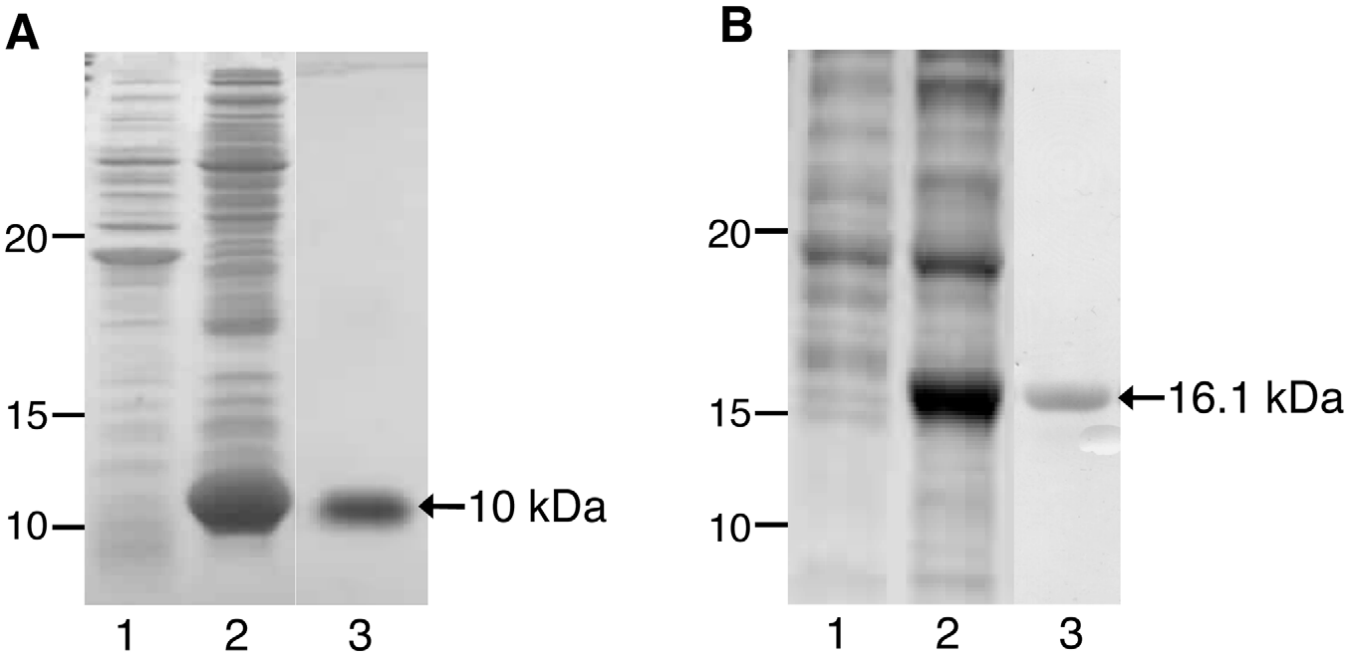
Vaccine antigen production. (A) *Sm*-TSP-2 was ligated to a pET41a vector backbone in-frame with the C-terminal 6×His tag, expressed in *E. coli*.by autoinduction and purified by IMAC followed by CEX chromatography and desalting into PBS. 1 = uninduced soluble fraction; 2 = autoinduced soluble fraction; 3 = purified protein. (B) *Sm*-TSP-2/5B was ligated to a pET41a vector backbone in-frame with the C-terminal 6×His tag, expressed in *E. coli*.by IPTG induction and purified by IMAC and desalting into PBS. 1 = uninduced soluble fraction; 2 = IPTG-induced soluble fraction; 3 = purified protein.

The large extracellular loop of *Sm*-TSP-2 (*Sm*-TSP-2) (molecular weight including 6×His tag = 10 kDa) was expressed in *E. coli* using the auto-induction technique of Studier [20] instead of the more conventional method of IPTG induction normally used to drive protein expression in T7 promoter-based, inducible systems. In addition to producing an increased biomass despite using identical seeding conditions and culture volumes, *Sm*-TSP-2 was produced by auto-induction and purified by IMAC to a final concentration of 100 mg/L (Fig. 1A), more than twice the yield of *Sm*-TSP-2 obtained by IPTG-induction (data not shown). To obtain reasonable yields of soluble chimeric *Sm*-TSP-2/5B (molecular weight including 6×His tag = 16.1 kDa), the protein required expression in the less reductive cytoplasmic environment of the slow-growing Rosetta-Gami strain of *E. coli*, in addition to being cultured at a sub-optimal growth temperature of 23°C; as a result, auto-induction of *Sm*-TSP-2/5B was not a feasible production method. Nevertheless, when expressed using IPTG-induction and purified by IMAC, we obtained a yield of 20 mg/L of soluble *Sm*-TSP-2/5B (Fig. 1B).

### Anti-*Sm*-TSP-2/5B antibodies bind to parasite-derived *Sm*-TSP-2 and neutralise the activity of *Na*-APR-1

**Figure 2:**
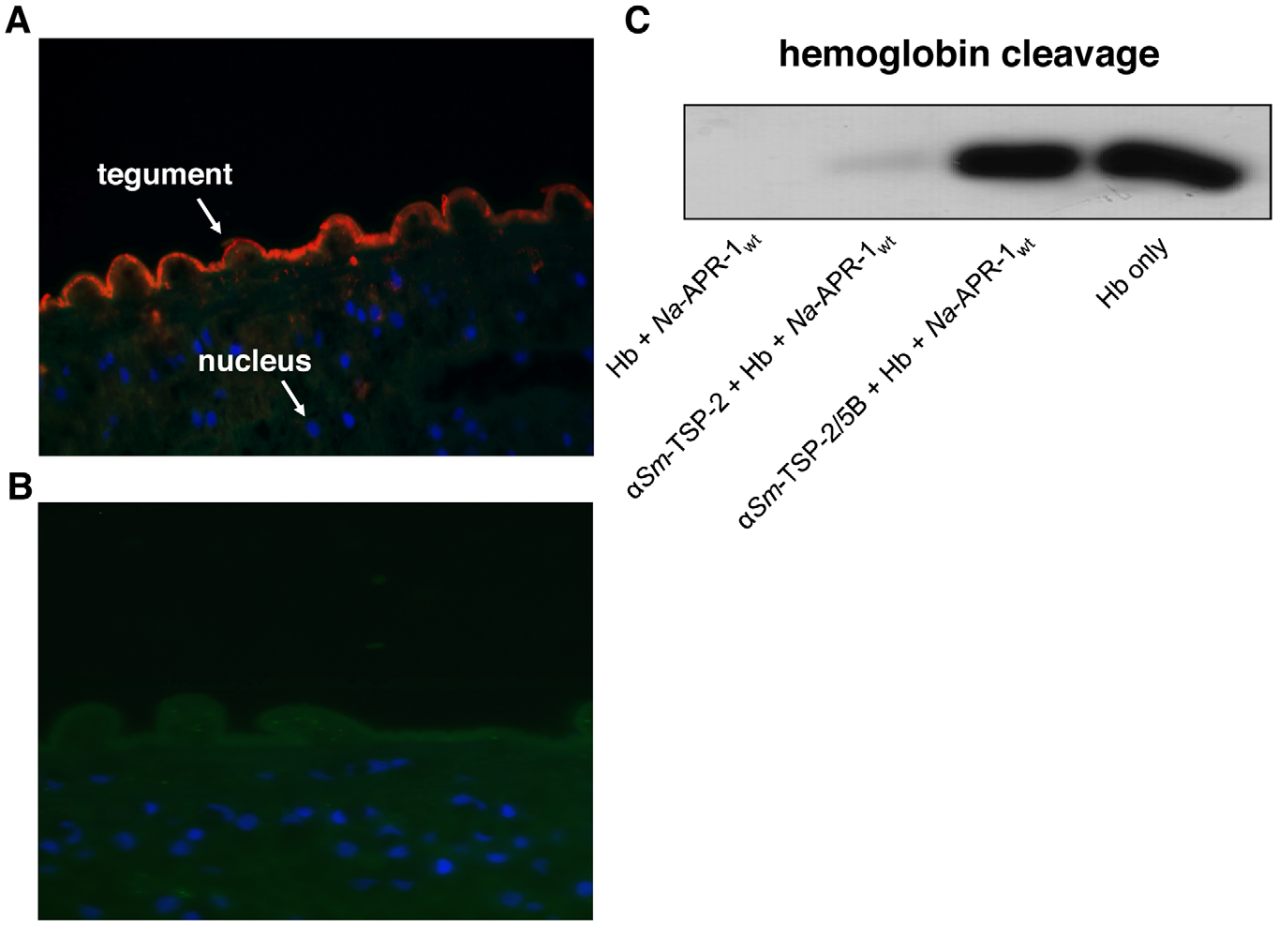
Anti-*Sm*-TSP-2/5B antibodies bind to parasite-derived *Sm*-TSP-2. Immunofluorescence micrograph showing adult male *Schistosoma mansoni* sections probed with either (A) rabbit anti-TSP-2/5B serum or (B) naïve rabbit serum followed by goat anti-rabbit IgG-Cy3. Both sections were also stained with Alexa-Fluor 488 (green staining of actin filaments) and DAPI (blue staining of nuclei). All images are shown at original magnification ×63.

The localization of *Sm*-TSP-2 to the outer tegument of *S. mansoni* has previously been documented using an antibody raised to the thioredoxin fusion protein [12]. The recognition of native *Sm*-TSP-2 by anti-*Sm*-TSP-2/5B antibodies (Fig. 2A) indicated that parasite-derived *Sm*-TSP-2 epitopes were faithfully reproduced in the recombinant protein and were not disrupted by the addition of the 5B region of *Na*-APR-1 to the C-terminus of *Sm*-TSP-2. No reaction was observed with naive rabbit serum (Fig. 2B). Similarly, the ability of anti-*Sm*-TSP-2/5B IgG to bind (and inhibit) *Na*-APR-1 hemoglobinase activity demonstrates the preservation of 5B epitopes within the chimeric protein. No hemoglobinase inhibition of the enzyme was observed when anti-*Sm*-TSP-2 IgG was used in the assay (Fig. 2C).

### Chronically infected individuals do not produce a detectable IgE response to *Sm-*TSP-2

**Figure 3:**
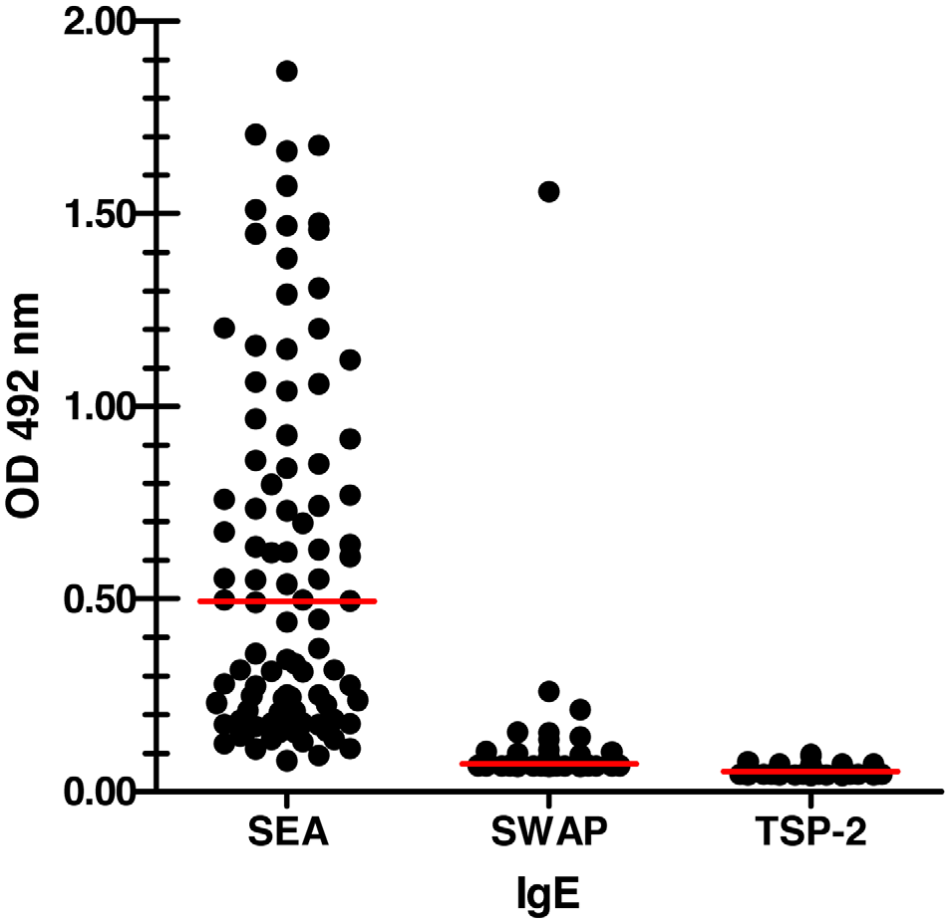
Chronically infected individuals do not make IgE against *Sm*-TSP-2. Graph showing human anti-*Sm*-TSP-2 IgE responses towards soluble egg antigen (SEA), soluble adult worm antigen (SWAP) and *Sm*-TSP-2. Antibody responses were determined by indirect ELISA. Dots reflect OD values of individual people and bars reflect the mean OD values of the groups.

Sera from 666 individuals from Minas Gerais state, Brazil – an area of high *S. mansoni* transmission – were assessed for the presence of an IgE response against *Sm*-TSP-2. No detectable levels of anti-*Sm*-TSP-2 IgE antibodies were observed, despite the presence of a strong IgE response to SEA in some individuals (Fig. 3).

### Antibody profile following mouse immunization and parasite challenge

Mice vaccinated with alum/CpG adjuvanted *Sm-*TSP-2 and *Sm-*TSP-2/5B mounted strong *Sm*-TSP-2-specific IgG responses (Table 2). IgG_1_ responses dominated and IgG_2a_ responses were generally weak (not shown). Pre-challenge IgG endpoint titers (four-fold serial dilutions) ranged from 256,000–1,024,000 for *Sm-*TSP-2 vaccinated mice and 256,000–4,096,000 for *Sm-*TSP-2/5B vaccinated mice. At necropsy (post-challenge), titers had waned to 64,000–256,000 for *Sm-*TSP-2 vaccinated mice and 64,000–1,024,000 for *Sm-*TSP-2/5B vaccinated mice. Mean and median anti-*Sm-*TSP-2 antibody titers were higher in the group vaccinated with *Sm*-TSP-2/5B (means 486,400 vs 1,450,667; medians 256,000 vs 1,024,000), implying that mice vaccinated with the chimera made a stronger antibody response against the *Sm*-TSP-2 region of the immunogen, and increased titers were not due to anti-5B antibodies. No obvious association between antibody titer and parasite burden was detected. Of the mice vaccinated with *Sm-*TSP-2/5B, two mice had no worms, one mouse had two worms and one mouse had four worms. All four mice had the lowest liver egg burdens and high antibody titers (≥1,024,000). However, two other mice had equally strong antibody titers but had higher parasite burdens (29 and 34 worms), precluding determination of a robust correlation between worm burdens and antibody titers.

**Table 2:** Anti-*Sm*-TSP-2 IgG titers and parasite burdens of *Sm*-TSP-2 and *Sm*-TSP-2/5B vaccinated groups from trial 1.

Mouse	Total IgG titer		Total Worms	Liver EPG^a^
	Pre-challenge	Necropsy		
***Sm-TSP-2 vaccinated group***				
1	256,000	64,000	4	14,652
2	256,000	64,000	38	18,722
3	256,000	64,000	30	16,061
4	256,000	64,000	44	18,543
5	1,024,000	256,000	53	16,473
6	256,000	64,000	48	20,430
7	256,000	256,000	25	4,813
8	256,000	64,000	38	13,900
9	1,024,000	256,000	56	16,250
10	1,024,000	64,000	28	11,293
***Sm-TSP-2/5B vaccinated group***				
1	256,000	256,000	32	11,217
**2**	**1,024,000**	**256,000**	**0**	**0**
3	256,000	64,000	38	17,296
4	256,000	256,000	51	16,952
5	1,024,000	256,000	34	13,523
6	4,096,000	256,000	29	9,832
**7**	**4,096,000**	**1,024,000**	**4**	**3,681**
**9**	**1,024,000**	**256,000**	**2**	**1,700**
**10**	**1,024,000**	**256,000**	**0**	**0**

a “EPG” = eggs per gram of tissue.

### Vaccination with *Sm*-TSP-2 and *Sm*-TSP-2/5B formulated with alum/CpG protects against parasite challenge

**Figure 4:**
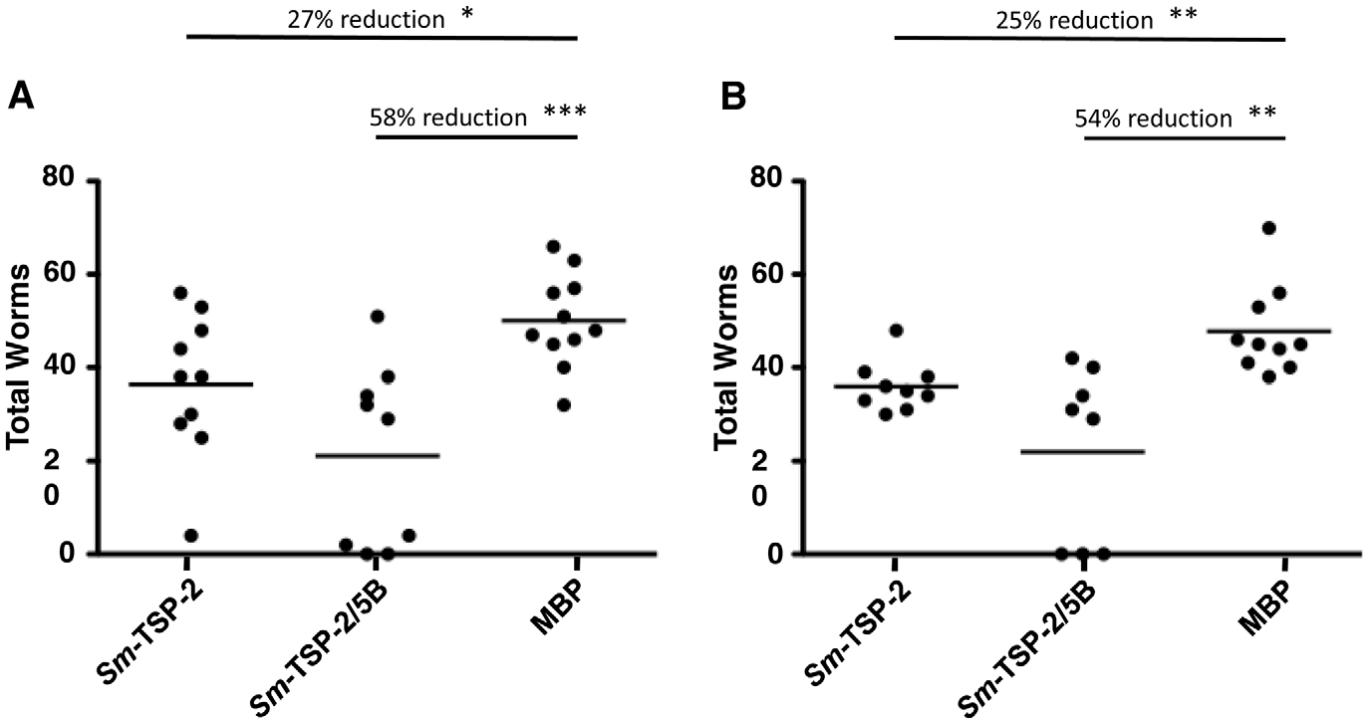
Worm burdens of vaccinated C57BL/6 mice. All vaccinees were necropsied on day 91, 7 weeks post-challenge. Worms were perfused from the vasculature with PBS into petri dishes and counted. Reductions and significance (*P<0.05; **P<0.01; *** *P*<0.001) are represented relative to the Maltose Binding Protein (MBP) control group. (A) Trial 1 total worms. (B) Trial 2 total worms.

**Figure 5:**
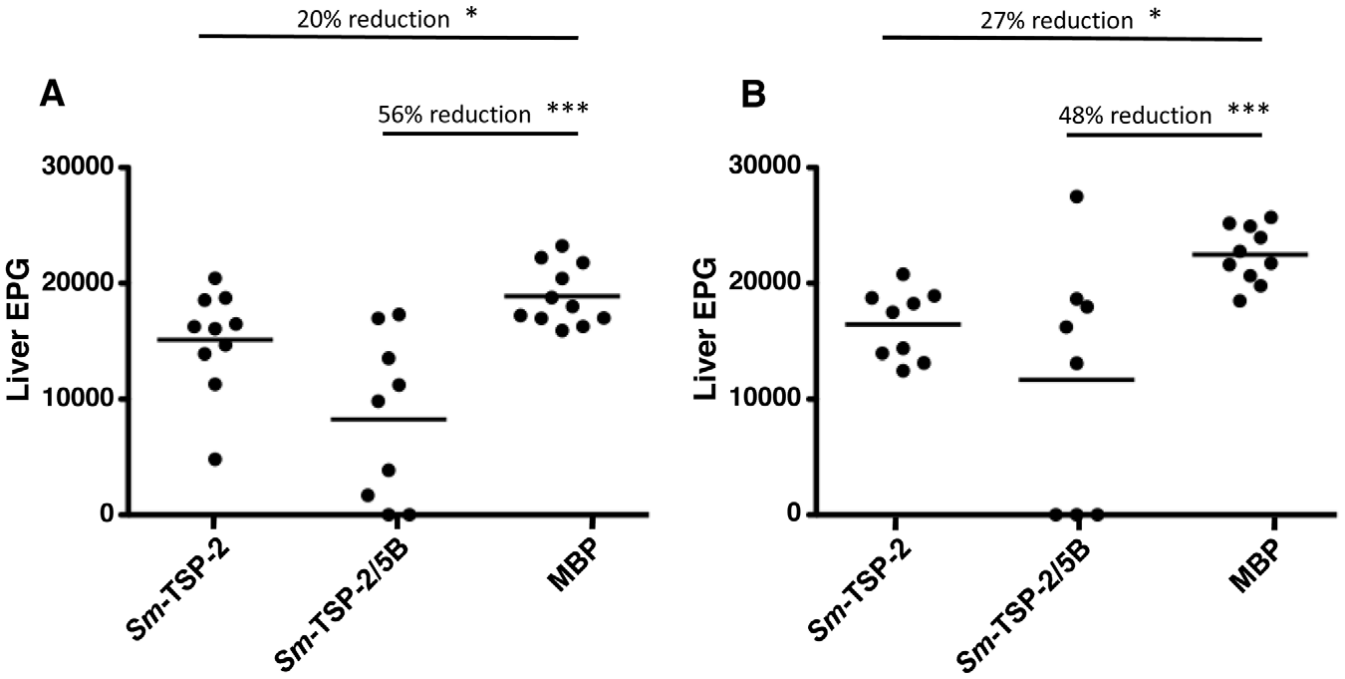
Liver egg burdens of vaccinated C57BL/6 mice. All vaccinees were necropsied on day 91, 7 weeks post-challenge. Livers were removed, weighed, digested in 5% KOH overnight at 37°C and the rsulting eggs resuspended in 1 ml formalin. The amount of eggs in multiple 50 µl aliquots were counted and the number of eggs per gram of tissue (EPG) determined. Reductions and significance (*P<0.05; **P<0.01; ***P<0.001) are represented relative to the Maltose Binding Protein (MBP) control group. (A) Trial 1 - liver EPG. (B) Trial 2 - liver EPG.

*Sm*-TSP-2/5B and *Sm*-TSP-2 formulated with alum/CpG protected against experimental challenge with *S. mansoni*. Vaccinated groups had respective decreases in worm burden of 54–58% (*Sm*-TSP-2/5B, *P*<0.01) and 25–27% (*Sm*-TSP-2, *P*<0.05), compared to controls over two independent trials (Fig. 4A and 4B). A comparative reduction in liver egg burdens was also observed in these groups – 48–56% (*Sm*-TSP-2/5B, *P*<0.01) and 20–27% (*Sm*-TSP-2, *P*<0.05), respectively (Fig. 5A and 5B). When the data from both trials were combined, significant decreases in worm and liver egg burdens were seen between the group vaccinated with *Sm*-TSP-2/5B and the group vaccinated with *Sm*-TSP-2 (*P*<0.01 and *P*<0.05, respectively). Liver egg burdens were not disproportionately reduced compared with burdens of worms, suggesting no additional effect on parasite fecundity (Table 2).

### Vaccine-induced protection against parasite challenge is associated with parasite-specific IFN-γ production

**Figure 6:**
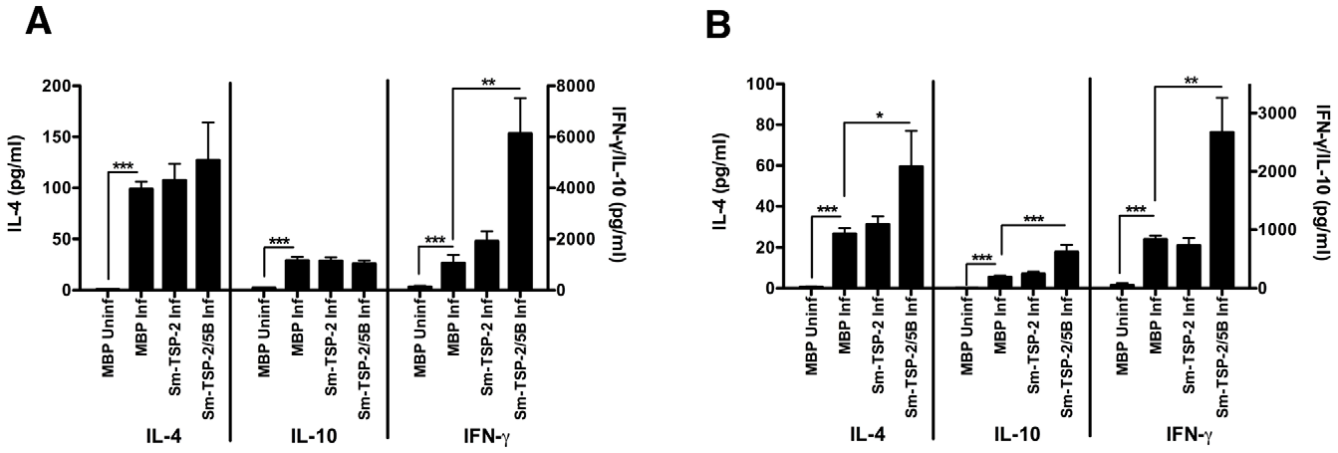
Protection against parasite challenge is associated with increased IFN-γ production. Graphs of ELISA data showing IL-4 (left axis), IL-10 (right axis) and IFN-γ (right axis) production by splenocytes taken from mice at necropsy and restimulated with either (A) schistosome soluble egg antigen (SEA) or (B) schistosome soluble adult worm antigen (SWAP). All significant differences are compared to the control MBP-vaccinated, infected group (MBP inf) by ANOVA (**P<0.01, ***P<0.001). Unless otherwise indicated, levels are not significantly different to controls.

Splenocytes from vaccinated and challenged animals were restimulated with SEA and SWAP to assess the cytokine responses to vaccination and parasite challenge. Levels of IL-4, IL-10 and IFN-γ from splenocytes were elevated in all infected animals compared to uninfected MBP-vaccinated animals when restimulated *ex vivo* with SEA and SWAP (Figure 6), indicating that infection-related cytokine responses were produced, although responses to SEA were generally higher. SEA and SWAP-specific IL-4 responses tended to increase in *Sm*-TSP-2/5B-vaccinated animals compared to control (MBP-vaccinated) infected animals, however this only reached significance with SWAP restimulation. IL-10 production in response to SWAP, but not SEA, was also increased due to *Sm*-TSP-2/5B vaccination. IFN-γ production in response to both SEA and SWAP were also highly significantly increased (P<0.01) in response to *Sm*-TSP-2/5B vaccination.

## Discussion

We have previously demonstrated that the large extracellular loop of the *S. mansoni* tegument tetraspanin, *Sm*-TSP-2, when linked to a thioredoxin fusion partner and formulated with Freund's adjuvants, is an efficacious vaccine antigen, eliciting high levels of protection in a murine schistosomiasis model of infection [12]. Herein, we show that modified and chimeric forms of the *Sm*-TSP-2 vaccine antigen are also protective, even when formulated with a human-approved adjuvant combination, and that a schistosomiasis vaccine based on *Sm*-TSP-2 (or *Sm*-TSP-2/5B) satisfies additional selection criteria for progression into clinical trials, such as safety concerns around the potentially deleterious effects of pre-existing IgE responses in helminth endemic populations [1], [19].

There is a paucity of funding - driven by the lack of a commercially viable market - available for the production of vaccines against the neglected tropical diseases, and so a vaccine antigen must be amenable to low-cost manufacture [22]. Despite attempts at optimisation of production of these two antigens being preliminary at best, both *Sm*-TSP-2 and *Sm*-TSP-2/5B have been expressed at yields that, at this initial stage, may be indicative of cost-effective up-scaling and clinical development. Indeed, *Sm*-TSP-2 has been recently produced in *Pichia pastoris* fermentation cultures in our laboratory at a yield or over 500 mg/L (data not shown) and efforts are currently underway to express *Sm*-TSP-2/5B in a similar fashion.

We recently suggested that the presence of a pre-existing human serum IgE response to a helminth vaccine antigen is a down-selection criterion [1] when considering a molecule for progression towards clinical trials because of the safety risks involved [19]. No detectable levels of *Sm*-TSP-2-specific IgE were found in individuals chronically infected with *S. mansoni*, despite very strong IgE responses to proteins found within SEA. This is also the case for the hookworm antigen, *Na*-APR-1 [23], the origin of the 5B domain in *Sm*-TSP-2/5B. Despite the absence of a detectable IgE response, previous studies have shown that humans from schistosome- and hookworm-endemic areas mount IgG_1_ responses to *Sm*-TSP-2 [12] and *Na*-APR-1 [23], indicating that both antigens are recognized by the immune system in a natural infection. What determines the isotype response (IgG vs IgE) mounted by an infected individual to a helminth antigen is multifactorial and an unresolved topic of debate [24]. What is clear, however, is the potential danger of developing a vaccine based on an antigen that is the target of a naturally acquired IgE response in the target population.

Of the two test groups, mice vaccinated with *Sm*-TSP-2/5B had the highest level of protection against experimental schistosomiasis. We initially hypothesized that this increased protection was due to cross-reactive epitopes within the 5B region of hookworm *Na*-APR-1 and its *S. mansoni* orthologue, *Sm*-catD [25]. However, numerous attempts to show binding of anti-*Sm*-TSP-2/5B to recombinant *Sm*-catD and schistosome extracts using Western blotting and immunoprecipitation coupled to tandem mass spectrometry (A. Dougall and A. Loukas, unpublished) were unsuccessful. Anti *Sm*-TSP-2/5B did, however, bind strongly to recombinant *Na*-APR-1 and inhibited the ability of the enzyme to cleave a synthetic substrate in a previous study [17] and has likewise been shown to neutralise the hemoglobinase capacity of *Na*-APR-1 in this study; indeed, the 5B region of *Sm*-TSP-2/5B is highly immunogenic and was the target of a panel of IgG_1_ mAbs raised to recombinant *Na*-APR-1 [17]. Production of a chimeric antigen comprising *Sm-*TSP-2 and the 5B region of *Sm*-CatD from *S. mansoni* instead of *Na*-APR-1 is currently underway in our laboratory and may have the additional benefit of being able to induce an antibody-mediated neutralization of *Sm*-TSP-2 in the tegument and *Sm*-CatD in the gut of the intra-mammalian stages of *S. mansoni*. Given the absence of an obvious cross-reactive schistosome epitope for antibodies to the *Na-*APR-1/5B fragment, we therefore sought to confirm whether the increased protection obtained with *Sm*-TSP-2/5B compared to *Sm*-TSP-2 alone was due to the increased size and therefore increased immunogenicity of the chimera. When microtiter plates were coated with *Sm*-TSP-2 and probed with antisera from mice immunized with *Sm-*TSP-2 or *Sm*-TSP-2/5B, the IgG endpoint titers were higher on average for the group immunized with *Sm*-TSP-2/5B, implying that vaccination with the larger immunogen resulted in an increased TSP-2-specific antibody titer. We also noted that individual mice with the highest antibody titers had the fewest worms, as highlighted in Table 2. Studies are also in progress to determine whether the chimeric protein generates similar levels of protection against hookworm infection caused by *Necator americanus*.

Restimulation of splenocytes from vaccinated and infected mice prior to necropsy showed a general increase in both Th2 (IL-4), regulatory (IL-10) and Th1 (IFN-γ) responses to parasite antigens, which was especially marked in increased IFN-γ production by mice vaccinated with *Sm*-TSP-2/5B compared to those vaccinated with the control non-parasite protein MBP. This implies that every animal was effectively challenged, indicating that the recovery of very few or no parasites in some mice was not due to an unsuccessful infection but successful vaccination. These data also suggest that Th1 cytokines have a role in the protective response against schistosomiasis, a finding that has been documented in infection studies with the parasite [26], [27] and vaccination experiments with recombinant vaccine candidate antigens from the tegument such as Sm29 [28] and Sm14 [29].

A caveat of using human-approved adjuvants to test vaccine antigens in the early stages of process development is that the full potential of a candidate antigen may not be realized due to the increased immunostimulatory properties of adjuvants containing mycobacteria and other toxic components, such as Freund's adjuvants. However, the levels of protection reported herein for *Sm*-TSP-2/5B were similar to those reported for Freund's formulated thioredoxin-*Sm*-TSP-2, and well exceed the 40% benchmark set by the WHO for progression of an antigen into clinical trials irrespective of the adjuvant used [30].

*Sm*-TSP-2 immunolocalizes to the surface of schistosomula [16] and adult worms [12] and has been found in the outer tegument of mature schistosomes [10] in abundance using proteomic techniques [31]. The ultrastructural morphology of adult worms and schistosomula treated *in vitro* with *Sm-tsp-2* double-stranded RNA displayed a distinctly vacuolated and thinner tegument compared to controls, suggesting that *Sm*-TSP-2 may play a pivotal role in tegument development in the early stages of intra-mammalian development [16]. These insights into *Sm*-TSP-2 function, along with the apparent importance of humoral immunity in anti-*Sm*-TSP-2 vaccination, lead us to hypothesize that the surface of the schistosomulum and adult fluke are potential sites of immune attack where these crucially important membranes are being opsonized by anti-*Sm*-TSP-2 antibodies for further attack by complement, antibody-dependent cellular mechanisms, or both. We are currently exploring the immunologic mechanisms responsible for vaccine-induced efficacy using genetically modified mice.

The *Sm*-TSP-2-based vaccine antigens reported in this study appear to exhibit all the early-stage characteristics of a vaccine targeting developing countries where schistosomiasis is endemic, based on their ease of production, absence of IgE reactivity, preferential recognition by resistant humans [12], essential nature of the protein for parasite survival [16] and vaccine efficacy in animal models. These features, coupled with the recent finding of a lack of polymorphism between geographical isolates of *Sm*-TSP-2 throughout Africa [32] provide a compelling argument for the use of *Sm*-TSP-2-based antigens as safe and effective anti-schistosomiasis vaccines. These additional studies also open the door to exploring more than a single helminth target with a single antigen.
